# CT diagnosis of giant lymph node hyperplasia in chest or abdomen

**DOI:** 10.1186/1471-227X-12-S1-A9

**Published:** 2012-12-18

**Authors:** CAO Jian-Xia, AO Guo-Kun, YUAN Xiao-Dong

**Affiliations:** 1 Department of radiology, The 309th Hospital of PLA,Beijing 100091, China

## Objective

To investigate CT characteristics of Castleman and their pathologic basis.

## Materials and methods

Thirteen cases of pathologically proved Castleman, encountered in our hospital during a period of five years, were collected in this study. The diagnoses were confirmed by surgery in all patients. The surgical, histopathologic records, and CT images were reviewed.

## Results

The lesion was localized in 5 and diffusely distributed in 3 cases. Pathologically, 5 cases belonged to hyaline vascular type and 3 cases belonged to plasma cell type, which 1 had retroperitoneal, 2 had mesentery and 5 had chest lesions. On CT scan, all cases showed round or ellipse soft tissue masses, with satellite nodules in 3 cases, and calcification in 3 cases. Enhanced scanning revealed obvious enhancement in the arterial phase and continuous enhancement in the vein and delayed phase in all the lesions, enlarged blood vessels within or around the mass were displayed in 3 case. In 3 cases, the intra-tumoral radial or fissured non-enhanced areas in early stage of enhancement were gradually filled up as the scan time was delayed.

## Conclusion

Castleman’s disease is rare and liable to misdiagnosis, a mass with benign characters, with calcification, especially delayed enhancement, can help in the diagnosis and differential diagnosis.

